# PROCare4Life lessons learned

**DOI:** 10.12688/openreseurope.16304.2

**Published:** 2024-03-22

**Authors:** Pilar Gangas, Elda Judica, Mayca Marin, Raquel Bouça-Machado, Joaquim J. Ferreira, Claudia Louro, Michael Brach, David Linnane, Mona Ahmed, Ellen Bentlage, Yusuf Can Semerci, Joao P. Proença, Jorge Alfonso

**Affiliations:** 1 INTERNATIONAL FOUNDATION FOR INTEGRATED CARE, The Base B Evert van de Beekstraat 1-104 Schiphol Airport, 1118 CBL, The Netherlands; 2 Casa di Cura Igea, Milan, Lombardy, Italy; 3 Association Parkinson Madrid, Madrid, 28014, Spain; 4 CNS - Campus Neurológico, Torres Vedras, 2560, Portugal; 5 KINETIKOS, Lisbon, 1700-093, Portugal; 6 University of Münster, Münster, 48149, Germany; 7 University Hospital Bonn, Bonn, 53127, Germany; 8 Maastricht University, Maastricht, 6211 LK, The Netherlands; 9 Universidad Politécnica Madrid, Madrid, 28040, Spain

**Keywords:** eHealth; Integrated Care; Lessons Learned; Digital technologies; People centred codesign; Parkinson’s; Alzheimer’s, dementia

## Abstract

PeRsOnalised Integrated CARE Solution for Elderly (PROCare4Life) was an EU-funded project that ran from January 2020 until June 2023, whose focus was to further develop and integrate previous ICT solutions developed by several previous EU-funded projects into a unique modular system able to support the autonomy and empowerment and to increase the Quality of Life (QoL) of elderly people living with Parkinson’s, Alzheimer’s, or similar dementia, having also tested the system for elderly people living with comorbidities. This article focuses on the methodology and results used to identify the internal lessons learned. PROCare4Life was developed using a codesign approach involving more than 2,000 participants whose input has been listened to and transformed into valuable changes of the system and also into lessons learned included in this case study report. Since the beginning of the implementation of PROCare4Life, there has been a commitment to make invisible knowledge visible through open discussion and including our lessons learned in each of our deliverables. In the last six months of implementation, qualitative research has been implemented by the PROCare4Life consortium to identify and select our most relevant challenges and recommendations for future projects and initiatives. PROCare4Life was highly impacted by the COVID-19 pandemic, and it is acknowledged in the lessons learned. However, the consortium has focused on the recommendations that could be more valuable for ordinary implementation of future projects and initiatives developing eHealth tools for elderly citizens living with conditions that might affect their cognitive or mobility capacities.

## Plain language summary

PeRsOnalised Integrated CARE Solution for Elderly (PROCare4Life) was an EU-funded project that ran from January 2020 until June 2023, aiming at improving the quality of life of older people living with Parkinson’s, Alzheimer’s or other dementia using ICT technologies. The term implementation refers to the process of putting a plan or idea into action. It is a complex process that involves multiple stages, including planning, execution, and evaluation. Implementation research is a growing field of health research that aims to study the factors that affect the implementation of health policies, programs, and practices. It can help identify the best strategies for introducing potential solutions into a health system or promoting their large-scale use and sustainability. ^i^ For PROCare4Life, when using the term implementation it covers all the stages, including ideation and design phases, although focusing on the pilot 3 iterative codesign and testing of the system. Using daily life devices such as smartphones and smart watches, more than 2,000 people have contributed to co-creating PROCare4Life. The three profiles focused on were older people living with Parkinson’s, Alzheimer’s or other dementia, their main carers and their healthcare professionals, with an ICT system providing direct communication and allowing them to share their health status. Along the journey to develop PROCare4Life, our European consortium has learned many things that we have internally investigated and reported in this article. We have identified 20 challenges and 41 recommendations. We hope that our lessons learned might be inspiring and valuable for others, particularly future projects and initiatives developing eHealth tools for elderly EU citizens living with different conditions that might affect their cognitive or mobility capabilities.

## Introduction

PeRsOnalised Integrated CARE Solution for Elderly facing several short- or long-term conditions and enabling a better quality of Life (PROCare4Life) has been an EU-funded project, implemented over the period of January 2020 through June 2023. The EU population is expected to change its age structure over the next few years, “turning increasingly grey”. ^ii^ The projected age-related expenditure is expected to be mostly driven by long-term care and health care spending. EU strong long-term care systems are expected to improve access to affordable and quality care, being the introduction of social and technological innovation expected to improve the efficiency of healthcare provision, enabling advancing on the integration of care. ^iii^ Digital health (eHealth) and care is aligned with EU Digital Strategy, aiming to use eHealth tools to improve access and quality of care, while increasing the efficiency of the health sector ^iv^, contributing PROCareLife to the three pillars including secure access and sharing, connecting and sharing health data for research, faster diagnosis and improved health and strengthening citizen empowerment and individual care through digital services. PROCare4Life is based on previous efforts and developments in other H2020 projects, including ICT4Life (ICT services for Life Improvement for Elderly), vCARE (virtual Coaching Activities for Rehabilitation in Elderly), mKinetikos, iWalkU, CrowdHealth, Heartman. Team members from 14 partners located in six EU countries had gathered to create, from a multidisciplinary perspective, the PROCare4Life solution This Innovation Action (IA) has aimed to contribute to the improvement of older adults' quality of life and better management of their condition, through an IT-based personalised, integrated care solution. PROCare4Life has been codesigned with its future end users; older adults living with chronic neurodegenerative conditions, Dementia, Parkinson’s and/or comorbidities, along with their informal carers and healthcare professionals. PROCare4Life sought to facilitate and improve monitoring and awareness, creating an Information and Communication Technology (ICT)-based support system allowing users to share their data with their selected caregivers and healthcare professionals. PROCare4Life developed a digital app that includes an easy-to-use personalised care plan and access to health and care professionals. Through wearable devices such as smartphones and Fitbit, together with other devices such as in-depth cameras and binary sensors in doors, the PROCare4Life system can monitor the user’s health data evolution, create personalised recommendations based on their Physical Activity (PA) or medication intake, and help older people and their carers to better monitor their health status, from an integrated, people-centred perspective. Artificial Intelligence (AI) is used to select the right information to be shared with healthcare professionals and to provide personalised recommendations to its users, thus supporting caregivers and promoting a better quality of life derived from adopting healthier habits, maintaining daily routines, and following personalised health advice provided by the PROCare4Life system, whose description has previously been published. User requirements were collected using mixed qualitative and quantitative research techniques that were iteratively tested and fine-tuned over the third phased approach to pilots’ implementation. Pilot 1 focused on testing the technical feasibility and usability of the PROCare4Life system. Pilot 2 focused on the characterisation and validation of the PROCare4Life system metrics. Pilot 3 focused on assessing the usability and clinical impact of the PROCare4Life final version, also addressing the replicability of usage of the system for other chronic conditions ^1^.

The main objective of PROCare4Life was to propose an integrated, scalable, and interactive care ecosystem which can be easily adapted to the reality of several chronic conditions, care institutions and end-user requirements, benefiting all the involved key stakeholders, elderly people living with Parkinson’s, dementia or comorbidities, their caregivers, their healthcare professionals and ultimately policy and decision makers. Its main contributions consist of the following:

-   Building an integrated scalable and interactive care ecosystem for neurodegenerative diseases and adaptable to other chronic conditions.

-   Finding the best actions/measures from a medical and social point of view that can facilitate an improved quality of life, awareness and care management for senior users suffering from neurodegenerative and/or other chronic conditions.

PROCare4Life is an integrated care solution, that has placed AI at the service of its users: detection of symptoms and signs, detection of deviations, detection of abnormal behaviour or data. Data is gathered for enough time to create an individual profile and thus identify when data or behaviour are deviant from usual. Personalised recommendations are derived from both the usual and unusual data and behaviour of end users. PROCare4Life has used both user centred design and codesign for developing its ICT system. Involving users along the development phases can be referred to as user-centred design ^v^ and it is expected to incorporate the needs of the future users and thus its usability and acceptance. ^vi^ When using the term "co-design", it is usually referred to the involvement of different stakeholders such as people living with conditions, their carers and healthcare professionals, to contribute to the development of new products since its very initial ideation phase ^vii^. The aim of this document is strictly linked to report PROCare4Life’s lessons learned, withthe pilots and phases of PROCare4Life implementation presented as contextual key factors only to frame our analysis. The results presented in this article are based solely on the contribution of the PROCare4Life consortium members. Final end users have not participated directly in this process.

## Methods

A lessons learned methodology is a process to identify, document, analyse, store, and retrieve valuable insights from projects. ^viii^ Lessons learned research has become one of the most relevant results for EU funded projects, aiming to capture the main challenges and best practices that can be of relevance for future initiatives. Lessons learned are focused mostly on the relevant knowledge derived from experience (positive or negative) in, projects ^2– 6^. Among the many lessons learned, a first criterion to be applied when prioritising those to be shared is that “the lesson must be significant in that it has a real value or assumed impact on operations; it should be valid – that is factually and technically correct and it should be applicable in that it identifies a specific action to be taken, replicated, avoided or adjusted”. From a methodological point of view, lessons learned derive from one of the most relevant methods used by the social sciences: case study. When identifying the lessons learned worth being shared, it is sought to uncover “the insights that might be generalised as constructive principles that suggest options to form productive future behaviours” ^6^. Thus, the main goal of the lessons learned approach is to learn from the past with the hope that it might positively affect the future, both carefully considering the challenges that can be confronted and using, as best as possible, the knowledge of what has worked best from previous experiences. The PROCare4Life project has been actively seeking to integrate knowledge from the testing and codesign fieldwork all over its implementation, thus developing a common aim to make invisible knowledge visible through internal inquiry, both during biweekly meetings of the consortium and including a subsection of lessons learned all over the project’s implementation. 

**Figure 1.**
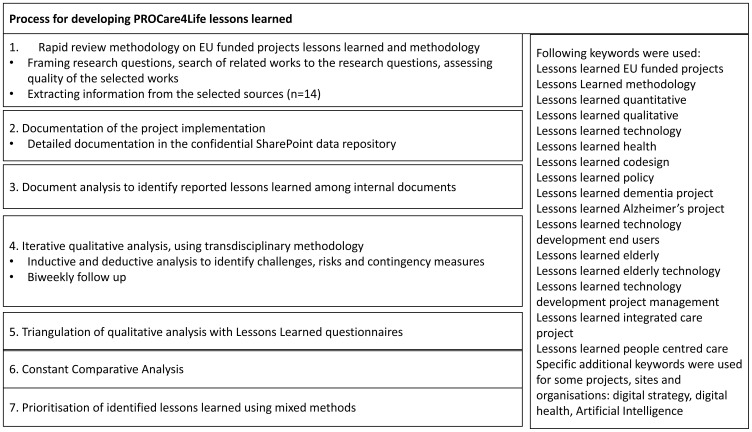
PROCare4Life Lessons Learned processual methodological approach. Source: Own elaboration.

Although PROCare4Life lessons learned have been researched across the project phases, only the lessons learned researched under Pilot 3 are described in the article. The methodology used across the project implementation has been an adaptation of the Project Management Institute (PMI) lessons learned methodology, from a processual perspective ^4^, as depicted in the flow chart (see Figure 1).

The methods depicted in the figure above are further described in the following paragraphs:

1. Using a rapid review methodology ^7^, identify previous methodologies and lessons learned results. Focusing particularly on previous EU funded projects, their public documents were searched, archived and analysed. ^ix^ The research questions were two: ‘What methodology have they followed to complete their internal lessons learned research?’ and ‘What lessons learned have they identified that could be of interest for PROCare4Life?’ We conducted searches over internet, using both Google Chrome and Bing, to identify previous projects and initiatives developing eHealth tools, particularly for targeted populations close to ours. A deductive process was used to identifying the keyword, including 16 keywords. CORDIS results from previous EU funded projects were also explored using these keywords. When identifying a particular project or organisation relevant to our research process, specific website visits for analysing the information were performed, of specific EU funded projects projects having shared their lessons learned or organisations sharing their lessons learned methodologies. Searches were repeated until the results that came out were no longer original. A deductive process was used to identifying the keyword, including 16 keywords, as summarised in Figure 1. CORDIS results from previous EU funded projects were also explored using the above keywords. When identifying a particular project or organisation relevant to our research process, specific website visits for analysing the information were performed, of specific EU funded projects projects having shared their lessons learned or organisations sharing their lessons learned methodologies. Searches were repeated until the results that came out were no longer original. The selection criteria for the relevant texts was based on the quality of the works and the connection with our own project objectives and lessons learned research questions. The next step involved extracting information from the selected articles, after critically appraising the information sources. Final rapid review involved information analysis and synthesis of the relevant sources explored ^x^ to identify both methodologies and lessons learned that could be applicable or transferable to PROCare4Life, including reporting styles for different targeted public. Relevant selected sources were explored in terms of their specific research methodology, lessons learned results ^8– 20^. Project Management Institute research methodology was adopted, among those explored, because of its clear processual approach. Among the identified lessons learned reporting styles, PROCare4Life team adopted a social research reporting style, considering it the most adequate for our target readers: future projects and initiatives, EU Commission experts, and policy and decision makers. ORE was identified as an excellent means to make our results visible.
2. Document: Detailed documentation of PROCare4Life implementation has been used as the basis for the lessons learned shared in this document and final events ( https://vimeo.com/831235005). The criteria followed to archive the information followed the internal general guidelines used by the consortium members, gathered in the confidential SharePoint repository of the PROCare4Life consortium in a specific folder called Lessons Learned.
3. Document analysis ^22^. PROCare4Life electronic documents were qualitatively searched for lessons learned, researching the preexisting textual sources. Results were organised using two dichotomic labels: challenges; recommendations. The sources included both the deliverables completed by the PROCare4Life consortium and the minutes from the biweekly meetings, that were discussed among consortium members.
4. Iterative qualitative method ^21^: a transdisciplinary methodology was developed by consortium members, combining both inductive and deductive analysis, that was completed periodically over the last 2.5 years of the project implementation. Biweekly meetings were used to identify challenges and contingency measures that were successfully implemented to manage risks, both those already expected and those unexpected (such as COVID 19). PROCare4Life has approached its own internal process of knowledge identification through this iterative, periodical internal sharing of challenges and solutions and its proper documentation of the lessons learned identified. Specific subsections have focused on reporting the lessons learned by PROCare4Life consortium members in the completed deliverables.
5. Triangulation of qualitative analysis was completed through the comparison of the previous information with the results provided by an internal questionnaire that was answered by PROCare4Life consortium members. Lessons learned questions included:
  a. What do you believe to be the lessons learned by PROCare4Life consortium that could be interesting for future initiatives?
  b. What would be your recommendations on things that should not be done according to our experience, for future initiatives?
  c. What were the key obstacles that PROCare4Life confronted?
  d. What do you think that has helped PROCare4Life to advance?
6. Constant Comparative Analysis ^23^: Qualitative analysis was performed comparing data and finding patterns, that allowed us to organise results in three different categories: end users’ related challenges and recommendations; ICT development related challenges and recommendations; and implementation related challenges and recommendations.
7. Prioritisation using mixed methods ^24^: On the list of identified lessons learned by category, consortium members polled those that they considered that should be prioritised. An internal workshop was used for the final prioritisations and fine tuning of the gathered lessons learned, thus creating a safe space for consensus creation whose results have been reported in this article.

Over PROCare4Life implementation, three waves of pilots in six pilots’ sites helped to codesign, fine-tune and improve the PROCare4Life system. The pilot sites have been the following: Spitalul Universitar de Urgenta Bucuresti (Bucharest); Association Parkinson Madrid (Madrid); Casa di Cura Igea (Milan); Campus Neurológico Senior (Lisbon); University of Medicine and Pharmacy (Bucharest); Wohlfahrtswerk für Baden-Württemberg (Stuttgart). These end users’ organisations have worked closely together with the technical partners developing the solution: Kinetikos (coordinators of PROCare4Life); Maastricht University (designing and developing the sensorial ecosystem), Universidad Politécnica Madrid (development of high-level subsystems), Software Imagination & Vision S.R.L. (generation of social and communication services), Atos (design of the integrated care platform). Other partners were the University of Münster (advising on the social sciences methodology of the users’ needs and requirements identification and generating physical activity recommendations as well as training materials for the users), International Foundation of Integrated Care (focusing on the validation of the integrated care approach and dissemination and communication) as well as Stelar (considering ethical and legal issues). All these organisations have contributed significatively to PROCare4Life lessons learned process, that is the focus of this article.

**Ethical considerations**: All the consortium partners have signed a data protection agreement, being the documents, this article is based upon classified as confidential by PROCare4Life Grant Agreement.

PROCare4Life large scale pilots for the iterative testing and codesign of the ICT solution obtained ethical approvals from the respective local Ethical Committees of each pilot site as follows.

(1) Wohlfahrtswerk für Baden-Württemberg: Approval from the Ethical commission of the University of Münster for the User-Requirements Study (2020-37-MB),

Pilot 1 (2020-59-MB-FA),

Pilot 2 (2021-15-MB-FA2),

Pilot 3 (2022-29-MB-FA4)

(2) Asociación Parkinson Madrid: Approval from the Ethical commission of Hospital Clínico San Carlos for the User-Requirements Study (20/453-E)

Pilot 1 (20/656-E)

Pilot 2 (21/220-E)

Pilot 3 – Clinical Study (22/392-E)

(3) Casa di Cura Policlinico: Approval from Comitato Etico Milano Area 2 of the Fondazione IRCCS Ca’ Granda Ospedale Maggiore Policlinico for the User-Requirement Study (ID Sperimentazioni 1796)

Pilot 1 (OSMANI-20/10/2020-0034210-U).

Pilot 2 OSMAMI-26/07/2021-0032326-U

Pilot 3 (OSMANI-22/09/2022-0044110-u)

(4) Campus Neurologico Senior: Approval of the Comissao de Ética Campus Neurologico Senior (BIO72685263)

Pilot 1 (N. Ref. 13-20)

Pilot 2 (N. Ref. 3-2021)

Pilot 3 (N. Ref. 6-2022-R)

(5,6) UHB and UMF, both located in Romania: Comisia de Etica e Cercatarii of the Spitalul Clinic Colentina.

Pilot 1 (Nr. 25/30.10.2020)

Pilot 2 (Nr. 24/28.09.2021)

Pilot 3 (Nr. 7/19.07.2022)

## PROCare4Life results

### PROCare4Life project results

**Figure 2.**
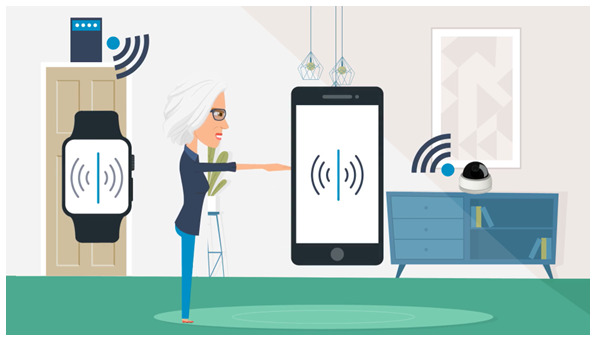
PROCare4Life system devices, home scenario.

The PROCare4Life solution includes elements and components to collect, process, store and output different types of information from and with the users. Before starting the pilots, needs by future end users were collected through devoted multidisciplinary research. The consortium consulted 217 participants to implement the user requirement study and the study protocol that has been published in JMIR ^25, 26^. Target users and scenarios were researched and described based on representative results and self-reflection summary with implications extracted from PROCare4Life. The design of the system adjusted to the most frequent or worrying symptoms referred by end users (stiffness 74%, feeling sad 67%, feeling anxious 64%, gait problems 62%, or loss of balance 62%) and their most desired functionalities (monitoring activities and symptoms 36%, information about physiological status 33%, communication with socio-health professionals 32%). A key requirement for the usability of the devices was for the system to be intuitive while nevertheless including training materials such as guides and manuals for users. These needs were turned into functionalities of the PROCare4Life system as follows: filtering and prioritisation of the most important symptoms for the participants (bradykinesia, festination, freezing, loss of balance, wandering); monitoring via sensors, wearables and cameras (symptoms detection, vital signs, sleep patterns, bathroom usage, activity patterns, steps, diary of symptoms); communication provided by PROCare4Life between patients, caregivers and healthcare professionals; Creation of reminders (medication intake) and professional alerts on symptomatology evolution (i.e., number of falls), together with lifestyle contents (physical exercise personalised recommendations); information about their conditions and ease of use by design of the PROCare4Life system and personalised training materials. Manuals were created for healthcare professionals using the web app, for patients and caregivers using the Android app, and for the patients using PROCare4Life Smart TV games on tablets or computers. Users were also provided with the Fitbit Versa 2 (Fitbit Health Solutions, San Francisco, USA) and its user manual for ease of handling. Figure 2 is a still from a promotional animated video used to invite participants, showing the system devices used in the home scenario.

Three pilot waves have been implemented, with the goal to codesign and iteratively check users’ feedback to continuously improve the system, according to its users’ preferences, thus putting people at the centre. Participants were continuously monitored, being key for the team to assure their positive perception regarding safety, comfort, usefulness of the system to facilitate their lives and thus increase their QoL despite the advance of their chronic conditions. Their feeling of empowerment was expected to derive from their increased perception of safety.

The three pilots involved 2,127 patients, caregivers, and healthcare professionals in the home, rehabilitation, and care home scenarios. Some positive responses were shared by participants after using the PROCare4Life system, such as feeling more informed, better monitored, and cared for, increased comfort and safety, and better communication with their healthcare professionals. However, it was possible to also identify some common challenges, monitored in the first two pilot waves. On the one hand, the installation and reinstallation of the devices that were to be included in the PROCare4Life system was considered challenging for the users, depicted in Figure 1: wristband, deep camera, mini-PC, smartphone or tablet, binary sensors for the doors, tablet, or computer for the cognitive games for patients and computer webapp for healthcare professionals. People living with dementia, Parkinson’s and/or comorbidities used progressively more finetuned versions of the PROCare4Life system at home, at rehabilitation rooms and in care homes. The devices were installed by the consortium team members, being really time consuming in its installation, calibration and learning soon that at the home scenario the participants were often not able to reinstall or recalibrate the system when required. It was also evident that the needs and acceptance of the different devices changed a lot across participant with the modularity of the system key to assure its personalisation. The amount of data gathered was another challenge during Pilots 1 and 2, and the consortium members accordingly decided to simplify the system, personalise it more, and increase the training that the users received, always according to their digital skills. A plug and play solution was agreed, interfaces were improved to increase usability and more personalised recommendations were also incorporated into the system. Some changes were also agreed on the backend.

**Figure 3.**
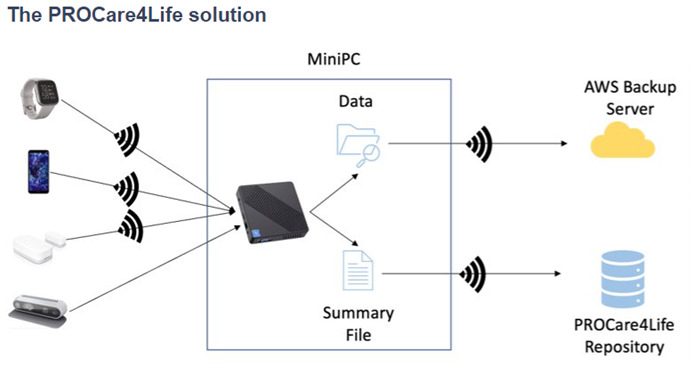
PROCare4Life data gathering and flow. PC solution is depicted in the Figure 4 below.

**Figure 4.**
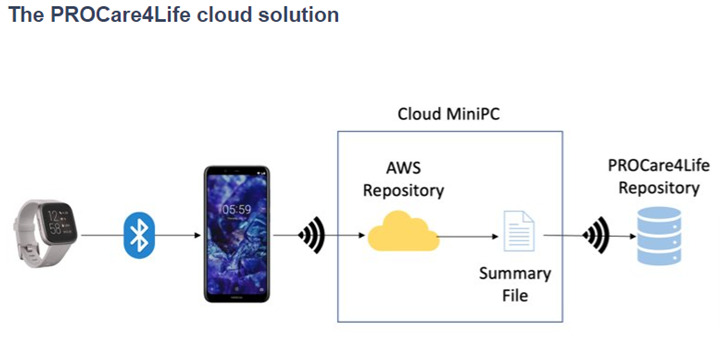
PROCare4Life system data gathering and data flow. Cloud solution.

Some of the patients were discouraged because of the technical problems in the first two waves of the pilots, and complained about not having access to their health data results, only available for healthcare professionals. Additionally, participants referred that they had to fill in too many questionnaires and disliked having to carry the smartphones around with them. Considering this and other additional feedback received from the different end users (also including carers and healthcare professionals), the PROCare4Life solution evolved from Pilot 1 to pilot 3. Significant implementation changes were carried out in response to the need to further reduce the complexity of the setup and usage of the PROCare4Life solution. Two different configurations were agreed, depending on the connectivity of each pilot site, the needs of the users and their preferences (see Figure 3 and Figure 4). It was also clear that a significant number of participants refused to use the in-depth cameras at their homes. In addition to a lower preference for the use of cameras in the home, one of the main reasons for non-use of cameras was that they could not be configured with the cloud version used in the homes, to simplify the procedure. There was also a problem with the camera licenses that was further considered when deciding on the below two configurations, particularly the cloud solution.

Biweekly meetings were held between pilot sites and technical partners to share the feedback received from the participants, to identify incidences and to solve them together. The recognition of user activities, including specific disease related symptoms were done using the technical tools included in the table below, each one connected with specific algorithms to detect the users’ daily activities and specific disease related symptoms (bradykinesia, falling, freezing, gait festination, loss of balance, wandering, normal). When data acquired were not large enough, synthetic data were created to feed the algorithms.

Thus, over 42 months, from a multidisciplinary perspective, the PROCare4Life Integrated Care Platform has been gradually codesigned and developed over time. One key lesson learned from Pilot 1 to Pilot 2 was to reduce the study duration from three to two months, to prevent dropout and fatigue of the participants. Additionally Pilot 2, included fewer and simplified questionnaires to survey seniors, also expanding the inclusion criteria to Parkinsonism. Finally, from the management of the recruitment, a more flexible approach was decided, reducing the sample for Pilot 2 and leaving a larger participation for Pilot 3 when the PROCare4Life system (see Table 1) was more technically mature and thus easier to use and test by participants. Weekly monitoring of the raw data was performed by the consortium members over pilot 2 and 3, together with the biweekly monitoring of patient recruitment. Additionally, preliminary analysis of data was performed biweekly to verify the amount and quality of the data export system, being considered by the consortium as one of our best practices, together with the iterative codesign approach.

**Table 1.** PROCare4Life symptoms, equipment, sensors and algorithms.

Activity Symptom	Equipment	Sensors	Algorithm
**Bradykinesia**	Smartphone	Accelerometer	Support Vector Machine (SVM)
**Falling**	Wristband + Camera	Accelerometer Skeleton Trajectories	Convolution Neural Network (CNN) + Long Short-Term Memory (LSTM)
**Freezing**	Smartphone	Accelerometer Gyroscope	CNN
**Gait Festination**	Smartphone	Accelerometer Gyroscope	Thresholding
**Loss of Balance**	Smartphone	Accelerometer	Autocorrelation
**Wandering**	Depth camera	Skeleton Trajectories from detected joints	LSTM Long-term thresholding
**Energy Expenditure**	Wristband	Optical heartrate sensor (OHS)	Heart rate grouping
**Heart rate**	Wristband	OHS	
**Inactivity**	Wristband	OHS	Sedentary minutes
**Indoor Mobility**	Door sensors	Binary sensor	Frequency
**Medication Intake**	Smartphone	-	Self-reports
**Sleep patterns**	Wristband	OHS Motion sensor	Thresholding
**Walking Patterns**	Smartphone Wristband	Accelerometer Gyroscope	Frequency

In all cases, the baseline of the participants was identified in their entry interviews, and that was repeated when exiting the study, thus allowing the consortium to compare indicators over time and thus identify variations. The exit process evaluated the participants’ acceptance (including perception of utility, usability, and willingness to use the system) of the different versions of the PROCare4Life system. Additionally, and in parallel, we researched the participants’ perception of the novelty and impact of the PROCare4Life system in the eHealth market and their predisposition to purchase it and to use it in the future, if available. The large scale of the pilots in the phase 3 required to implement varied and adapted recruitment strategies, including online webinars, contacting patients’ associations, delivering brochures, social media campaigns, mail campaigns, promotion at local pharmacies, and assuring the adequate visibility of the project in different specialised Conferences.

### 3.2. PROCare4Life Lessons challenges and recommendations

Our 20 key challenges and 41 recommendations are presented in this section. Lessons learned have been organized under several large categories: challenges and recommendations related to: Implementation, End Users; Technical, as depicted in Table 2.

**Table 2.** Types of challenges and recommendations.

Challenge/Recommendation type	Meaning
**IMPLEMENTATION**	Unforeseen challenges or successful contingency measures implemented related with the implementation of the project
**END USERS**	Unforeseen challenges or successful contingency measures implemented related to the pilot end-users (Healthcare Professionals, Patients and Caregivers)
**TECHNICAL**	Unforeseen challenges or successful contingency measures implemented related to the technical implementation of the project

Table 3 summarises the main challenges that the PROCare4Life consortium has identified, being the limitations derived from COVID-19 relevant for the initially planned implementation of the project. On the technical and end users’ dimensions, there were some unexpected challenges linked to the data collection, installation and calibration of the devices and usage of off the shelf technologies and the need to acknowledge that training for both patients, caregivers and healthcare professionals was required.

**Table 3.** PROCare4Life challenges.

#	Challenge Type	Challenge Short Description	Challenge Explanation
1	IMPLEMENTATION	COVID-19	COVID-19 pandemic disrupted the usual procedures in the pilot sites’ organisations, making recruitment challenging for a long time. Additionally, increased mortality among the target users resulted in reluctance among end- users to enter the premises of the pilot sites or allow project staff into their homes.
2	IMPLEMENTATION	Heterogeneity of the end users’ organisations involved	Each site and country had its own respective country, regional or organisational policies, resulting in a great heterogeneity of the centres involved in terms of the samples of subjects and technical capacities. These variations across sites created additional challenges during the implementation of PROCare4Life, requiring coordination and adaptation efforts.
3	IMPLEMENTATION	Large scale pilot recruitment	The large-scale pilots involved significant challenges linked to the recruitment for the pilots, making the project more demanding without translating into clear benefits for codesign and iterative testing purposes.
4	IMPLEMENTATION	Unforeseen costs and tasks	The Grant Agreement had to be interpreted and operationalised, andsome costs were not forecasted, as those linked to the re-use of the previous technologies coming from previous EU funded projects, or the time required for data monitoring, analysis, and reporting.
5	END USERS	Low technology literacy among participants	Expected active participation of the participants in their interaction with technology appeared to be overly optimistic. Low ICT skills were detected early among participants.
6	END USERS	Saturation of participants due to numerous clinical scales	Participants were required to complete a high number of clinical scales as part of the initial study protocol. This extensive workload led to saturation and potential challenges in maintaining participant engagement and compliance, especially for individuals with limited mobility or cognitive impairments.
7	END USERS	Number of technological devices for participants	PROCare4Life was initially planned for participants to interact with several technological devices simultaneously. However, the number of devices was considered excessive by participants, who found it difficult to manage, charge, update, calibrate and navigate multiple devices concurrently. This affected the overall user experience and potentially led to reduced compliance.
8	END USERS	Reluctance to use cameras at home due to privacy concerns	Participants expressed concerns and resistance to using depth cameras at home due to privacy fears and the potential risk of being recorded. This hesitation created a barrier to the adoption of this technology, requiring alternative solutions or modifications to ensure participant comfort and trust in the system.
9	END USERS	Healthcare professionals understanding of the PROCare4Life system	Health professionals responsible for onboarding patients in the study required comprehensive training to ensure their understanding of the system's functionalities and their ability to effectively guide and assist participants. Adequate training programs had to be developed and implemented to address this challenge.
10	TECHNICAL	Integration of data from multiple devices	The project required collecting and correlating data from several devices with different APIs and communication protocols. Managing the integration and synchronization of data from these diverse devices posed technical complexities, requiring specialized development and coordination efforts.
11	IMPLEMENTATION	Need for PC installation at the site, especially in home scenarios	The study required the installation of devices at the sites, particularly in home scenarios, that implied increased logistical complexities and unforeseen high resources allocation.
12	END USERS	Non-technical personnel installing equipment	Non-technical personnel were responsible for installing the equipment, which introduced challenges in ensuring accurate and effective installation and setup procedures. Additional training and support had to be provided to non-technical personnel to minimize the risk of errors or system malfunctions during the installation process, and to establish quicktechnical support from technical team members to team members from pilot sites in charge of installation, monitoring, and calibration of devices.
13	IMPLEMENTATION	Equipment availability and adaptation	The project faced challenges due to the unavailability of certain devices (e.g., smartphones, depth cameras) during the project's timeline. Additionally, the need to adapt the system between different pilots rendered some previously purchased devices irrelevant. These unexpected limitations and adaptations required new equipment purchases and adjustments, impacting the project's budget and timeline.
14	TECHNICAL	Dependency on brand- specific technologies	The project relied on technologies that were specific to certain brands or proprietary systems. This brand dependency introduced challenges in terms of compatibility, interoperability, and potential vendor lock-in, which could limit flexibility and scalability in the long run.
15	TECHNICAL IMPLEMENTATION	Refinement of technologies from previous projects	Technologies used in previous academic projects required unexpected adjustments and refinements to make them suitable for large-scale pilots. These adaptations posed technical challenges and necessitated additional development efforts to ensure the successful reuse of results from the previous projects.
16	TECHNICAL	Unplanned factory updates	Unannounced and unexpected updates from equipment factories disrupted the functioning of the system. These updates often necessitated immediate adjustments and modifications to ensure compatibility and stability, causing interruptions and potentially impacting the reliability and performance of the system during the pilots.
17	TECHNICAL	Ongoing system maintenance and updates	The multi-year project duration necessitated regular system maintenance and updates. Technological advancements and evolving industry standards required periodic updates to components such as the application (app) to ensure compatibility with new devices, operating system updates, and emerging technologies. These maintenance efforts were essential but initially underestimated in terms of time and resources.
18	IMPLEMENTATION	Time allocation for data analysis and adjustments	Adequate time for comprehensive data analysis and adjustments between pilot phases and at the end of the final pilot was not initially accounted for in the project timeline. The complexity and volume of data collected, combined with the need for in-depth analysis, required additional resources and extension of the project's duration.
19	IMPLEMENTATION	Inability to hold in-person consortium meetings	The ongoing COVID-19 pandemic prevented most in-person consortium meetings from taking place during the project's implementation. The absence of face-to-face meetings limited direct collaboration, communication, and decision-making among consortium members, introducing additional challenges in project management and coordination until in person meetings were possible.
20	IMPLEMENTATION	Time for scientific publications once results are available	The communication, dissemination and impact strategy need to be closely connected with the different implementation phases of the project, having limited time to make results available through peer reviewed scientific publications.

In response to the challenges included in the previous table, PROCare4Life consortium looked for contingency measures to successfully manage them. In Table 4 it has been included the main PROCare4Life recommendations.

**Table 4.** PROCare4Life recommendations. Column 1: number of the recommendation, column 2: related challenge from Table 3.

#	Connected Challenges	Recommendation
1	1 COVID 19 IMPLEMENTATION	Flexibility in the methodological approach when required. To cope with COVID-19 limitations, the consortium adapted the methodology and planning of the pilots, reducing them during COVID and enlarging the number of participants for later stages. Internally, communication among consortium members was strengthened with frequent meetings to support multidisciplinary teamwork. Additional, flexible installation options were agreed. This involved providing self- installation kits with detailed instructions and remote assistance for troubleshooting and reducing the number of participants when the situation is challenging, to increase them in later stages.
2	1 COVID 19 IMPLEMENTATION	Increased communication about the project to support participation. Establish comprehensive project information and training activities for participants. It might involve communicating accurate information about the project's objectives, benefits, and safety measures directly to them. Engaging healthcare professionals in the recruitment process to provide reliable information to patients and caregivers can also enhance confidence among potential participants.
3	2 Heterogeneity of the end users’ organisations involved. IMPLEMENTATION	Increased internal communication among consortium members is key for managing risks, establishing common guidelines and protocols for successfully implementing contingency plans across different sites. Clear communication and collaboration dynamics should be promoted from coordinators, allowing consortium members to discuss and find the successful strategies and learn from each other's experiences with transparency.
4	2 Heterogeneity of the end users’ organisations involved. IMPLEMENTATION	Conduct pre-implementation comprehensive site assessments to identify site-specific challenges and requirements. This assessment should consider factors such as local regulations, available resources, and potential limitations. By understanding the unique needs of each site, future projects can proactively address potential variations in contingency plans, plan appropriate adaptations, and allocate necessary resources accordingly. Regular communication with site administrators and stakeholders throughout the project can also help identify and address emerging challenges promptly.
5	3 Large scale pilot recruitment. IMPLEMENTATION	If implementing large scale pilots, make sure to conduct feasibility studies and pilot tests in lab conditions before bringing the technology to real life environments. Flexible allocation of participants, that should be increasing according to the level of development of the technology, would allow to take full advantage of the participants’ contribution. Be creative and make sure to be flexible enough to allow different profiles of participants, with different level of commitment, to have the means to participate in your project.
6	3 Large scale pilot recruitment. IMPLEMENTATION	People-centred technologies require putting people at the centre. It is never too early to involve real people in the development of eHealth technologies. The integrated care approach has proven useful: the integrated care approach combining the integrated care pathway, technology integration and change management.
7	3 Large scale pilot recruitment. IMPLEMENTATION	Make sure that the recruitment and testing results are monitored at least biweekly and that the feedback from participants is shared among both technical and end users’ organisations, discussing at least biweekly the improvements to be implemented in the technology being developed.
8	4 Unforeseen costs and tasks IMPLEMENTATION	Prioritize thorough and detailed discussions during the grant agreement negotiation phase to minimize ambiguities. Seek clarification on potential gray areas, unforeseen costs, and tasks to ensure a more comprehensive understanding of the project scope and requirements.
9	5 Low technology literacy. END USERS	Training is a must for all end users. Include it in your planning from the very beginning, personalised by profile of end users and technology to be used, using multimedia, intuitive means to communicate the information required to manage devices. Consider the digital skills of future users when designing the system, ensuring it is user-friendly and accessible to all skill levels.
10	6 Too many scales END USERS	Prevent participant fatigue by limiting the number of clinical assessments, prioritizing essential measures, and minimizing the burden on participants. Make sure to test the time required for initial and exit interviews, responding to questionnaires and scales, identifying early if the time and language are adapted to your target population. Fatigue among elderly people living with different conditions require testing it before starting the actual pilots, to prevent their saturation and dropout.
11	7 Number of devices. END USERS, IMPLEMENTATION	If your target population is people living with cognitive or physical impairment, particularly elderly people with limited digital skills, make sure to design a system as simple and automatic as possible. Design a system that is more passive and minimalist, making it user-friendly and less overwhelming for participants. Keep the system as simple as possible, minimizing complexity and user requirements to enhance usability and user acceptance.
12	8 Reluctance to use in depth cameras. END USERS	Involve users in the co-design process from the very beginning, incorporating their feedback and preferences to ensure the system meets their needs. If they feel uncomfortable with biometric cameras or any other device at their homes, make sure to find an alternative approach that respects their wishes, always supporting their autonomy and empowerment to manage as much as possible their own conditions.
13	8 Reluctance to use in depth cameras. END USERS	Ensure that the system provides clear and personalized benefits for users, tailored to their respective needs, to increase acceptance and adherence.
14	9 Healthcare professionals understanding the system. END USERS	Tailor the system closely to users' preferences and needs, considering their feedback and requirements throughout the design and implementation phases. Make sure to understand the needs and wishes of healthcare professionals from the very beginning, together with their digital skills. They are key stakeholders for the success of any eHealth technology and thus it is key to make sure that they understand the system.
15	5 Low digital literacy, END USERS, IMPLEMENTATION	Plan sufficient time for training the participants, ensuring they have a thorough understanding of the system and its functionalities.
16	7 Number of devices. END USERS, IMPLEMENTATION	Select only one or two devices, using a modular approach for technology testing and deployment, reducing complexity for both users and implementation.
17	10 Integration of data from different devices. TECHNICAL	Avoid overlapping different pilots and adopt a sequential approach to technology testing, allowing for focused evaluation and effective implementation.
18	10 Integration of data from different devices TECHNICAL	Implement a phased approach to technology testing, conducting feasibility and validation in controlled environments before involving real participants. Conduct pre-piloting periods in controlled environments (lab conditions) to test the technology's feasibility and validate its performance before involving real participants. Ensure that technical solutions are thoroughly perfected and tested before large-scale deployment, minimizing issues, and optimizing system performance.
19	12 Non-technical personnel installing equipment. TECHNICAL	Match each pilot end-user organization with a technical support partner, providing dedicated assistance and troubleshooting during the pilot phase.
20	13 Equipment availability and adaptation. TECHNICAL	Anticipate decisions on devices and consider joint purchases, ensuring compatibility and streamlined deployment across different pilots.
21	16, 17 Unplanned factory updates and ongoing system maintenance and updates. TECHNICAL	Establish a rapid response mechanism to address technical problems promptly, minimizing disruptions to the system's functionality and user experience.
22	10 Integration of data from multiple devices TECHNICAL, IMPLEMENTATION	Promote a Continuous Development/Continuous Implementation Strategy to maintain a continuous flow of communication between technical and clinical teams, ensuring seamless integration and progress throughout the project.
23	10 Integration of data from multiple devices TECHNICAL	Explore alternative data collection methods: Future projects should consider alternative data collection methods that reduce the reliance on PC installations at the sites, particularly in home scenarios. This can involve leveraging mobile devices, such as smartphones or tablets, as data collection tools. Developing mobile applications or utilizing existing communication apps can facilitate remote data collection, reducing logistical complexities and resource allocation associated with PC installations.
24	11 Need for PC Installation at the site, especially in home scenarios. TECHNICAL	Streamline installation processes and logistics: To address the logistical complexities related to PC installations, future projects should develop streamlined installation processes. This can involve providing detailed installation guides and pre-configured equipment to simplify setup procedures. Additionally, establishing partnerships with local IT service providers or leveraging remote installation and troubleshooting technologies can expedite the installation process, ensuring timely and efficient deployment of the system.
25	11 Need for PC Installation at the site, especially in home scenarios. TECHNICAL	Avoid relying on self-installation by end users with cognitive or physical impairments, particularly elderly individuals living with long-term conditions.
26	13 Equipment availability and adaptation. TECHNICAL, IMPLEMENTATION	Conduct regular technology assessments: Future projects should conduct regular technology assessments to identify potential equipment availability challenges and anticipate emerging needs. By staying informed about technological advancements and market trends, projects can proactively plan equipment purchases and adapt to evolving requirements. Engaging with industry partners and suppliers can provide insights into upcoming technology releases and facilitate the procurement of devices that align with the project's long-term objectives.
27	13 Equipment availability and adaptation. TECHNICAL, IMPLEMENTATION	Establish contingency plans and flexible budgets: Given the unpredictable nature of equipment availability and the need for system adaptation, future projects should establish contingency plans and flexible budgets. Allocating a portion of the budget for unforeseen equipment purchases and adjustments can mitigate the impact of unexpected limitations. Additionally, maintaining open communication with funding agencies and stakeholders throughout the project's lifecycle can facilitate timely budget adjustments and ensure financial flexibility to address equipment-related challenges.
28	17 Ongoing system maintenance and updates. IMPLEMENTATION	Allocate budget for purchasing SIM cards in pilots where connectivity is limited, ensuring reliable and uninterrupted data transmission throughout the project.
29	19 Inability to hold in-person consortium meetings. IMPLEMENTATION	Foster multidisciplinary enhanced communication among consortium members to facilitate collaboration and knowledge sharing across different domains.
30	15 Refinement of technologies from previous projects. TECHNICAL IMPLEMENTATION	Conduct a thorough assessment of technologies from previous academic projects to evaluate their scalability and readiness for large-scale pilots. Consider conducting additional testing and refinement before implementing these technologies to ensure their suitability for the specific project requirements.
31	15 Refinement of technologies from previous projects. TECHNICAL IMPLEMENTATION	Provide legal and ethical support throughout the project, ensuring compliance with regulations and ethical standards related to data protection and privacy.
32	15 Refinement of technologies from previous projects. TECHNICAL IMPLEMENTATION	Foster collaboration between academic researchers and industry professionals to bridge the gap between academic projects and real-world implementation. Encourage joint research and development initiatives to refine and adapt academic technologies for practical applications, making them more suitable for large-scale projects.
33	16 Dependency on brand-specific technologies. TECHNICAL	Establish a close relationship with equipment manufacturers and stay informed about potential updates or changes to their products. Engage in proactive communication with the manufacturers to receive early notifications about upcoming updates, allowing the project team to plan and prepare for necessary adjustments in a timely manner.
35	16 Dependency on brand-specific technologies. TECHNICAL	Develop a robust system architecture that is flexible and adaptable to accommodate unexpected updates. Implement mechanisms that allow for seamless integration of new updates without disrupting the overall functioning of the system, minimizing the impact on the project timeline, and ensuring the stability of the technology used.
36	17 Ongoing system maintenance and updates. TECHNICAL	Develop a comprehensive maintenance and update plan from the outset of the project. Allocate dedicated resources and time for regular system maintenance, including periodic updates to software, hardware, and compatibility with emerging technologies.
37	17 Ongoing system maintenance and updates. TECHNICAL	Stay updated on technological advancements and industry standards throughout the project duration. Establish a process for monitoring and evaluating new developments, ensuring the system remains up-to-date and compatible with the latest devices and operating systems. Regularly assess the impact of new advancements on the project and allocate resources for necessary adjustments and updates. Specify and demonstrate data protection and privacy measures by design and implementation of the platform, ensuring compliance with relevant regulations and standards.
38	19 Inability to hold in person consortium meetings. IMPLEMENTATION	Foster transparency of information and effective communication among consortium members to identify and resolve common challenges and find collective solutions when in person meetings not possible, creating a culture of multidisciplinary collaboration to support teamwork. As soon as possible when feasible, increase the understanding among team members through in-person consortium meetings, promoting effective collaboration and alignment throughout the project.
39	20 Time for scientific publications once results are available. IMPLEMENTATION	Be aware of the time required for publications and plan accordingly to disseminate project findings and outcomes within the specified timeline.
40	20 Time for scientific publications once results are available. IMPLEMENTATION	Establish shared procedures for publication, encouraging collaboration and knowledge sharing among consortium members for efficient dissemination of project results.
41	20 Time for scientific publications once results are available. IMPLEMENTATION	Initiate the exploitation-related work as soon as possible, fostering familiarity with the exploitation strategy within the consortium for effective project outcomes.

After the internal prioritisation process was completed, the key lessons learned from PROCare4Life for the future research projects developing eHealth digital tools have been identified and summarised (see Table 5– Table 7).

**Table 5.** PROCare4Life prioritised recommendations related to end users.

Recommendations related to end users
1. Make sure to consider the digital literacy of users when designing your testing
2. Tailor your system to the real user’s preferences and needs, involving them in the development process from the very beginning and all along the process. Make sure to provide personalised feedback to participants and that the system gives back to those using it for increased acceptance and adherence
3. Limit the number of clinical scales to be passed to each participant to prevent fatigue, particularly for participants living with dementia
4. Plan and budget training for your users. Training materials need to be personalised and make sure to plan time for training your participants
5. Make sure that the system has a modular approach that might adjust to the respective wishes and needs of the end users.

**Table 6.** PROCare4Life Prioritised technical related recommendations.

Technical related recommendations
1. Keep the system as simple and automatic as possible. Prevent systems that involve self-installation requirements
2. Make sure to grant a rapid response to the technical problems
3. Integrating data and devices, particularly if coming from other previous projects or off the shelf, might be less smooth than expected. Plan additional time and look for credible alternatives to the initial plan when required
4. Target large scale testing only when the system works properly, to prevent frustration from participants
5. Make sure to have a Plan B for connectivity issues, devices management and brand of devices

**Table 7.** PROCare4Life Prioritised implementation related recommendations.

Implementation recommendations
1. Make sure to manage the unforeseen activities and costs as early as possible, particularly grey areas in the Grant Agreement
2. Internal communication is key for the project, make sure to create continuous communication considering multidisciplinary and cultural differences
3. Incorporate Data Protection and Privacy by design in your IT eHealth system
4. In person consortium meetings are very important to complete the transition from being a group of individuals to being a team
5. Consider the heterogeneity of the pilot sites to adapt the implementation methodology

## Discussion

We described a complex AI project in the field of integrated care. Based on experiences with the three pilots, involving 2,127 elderly people living with Parkinson’s, Alzheimer’s or other similar dementia, caregivers, and healthcare professionals, we used the lessons learned methodology to identify 20 challenges, grouped them into three categories (implementation, end users, technical) and formed 41 recommendations, which may be useful for future projects.

The transition from RIAs (Research and Innovation Action) to IAs (Innovation Action) under Horizon 2020 might be less smooth than expected. Several dimensions of challenges might make it difficult to reuse previous EU funded projects results, such as IPR (Intellectual property) rights of organisations that are not participating in the new consortium, organisations that have vanished and no person can explain how the previous system worked, interoperability between previous solutions. In that sense, favouring more realistic, less ambitious projects, could be another lesson learned to be highlighted.

The lessons learned methodology has proven useful to identify the key challenges that PROCare4Life has confronted and to make sure that some of the contingency measures adopted can be useful for future projects. Challenges with respect to implementation, technical and end users’ dimensions have been properly-managed thanks to developing a multidisciplinary collaboration internal procedures and culture, based on constant mutual support to make sure to incorporate the feedback coming from our participants, thus bringing forward a truly people centred, integrated care technology.

Change management for the correct integration of the PROCare4Life solution will involve additional iterative testing, but also additional work on the integration of the system into the current care pathways to promote the integration of care and quality of life of patients, carers and healthcare professionals using the system.

Most challenges and recommendations reported here address the project consortium. However, it is worth to contribute to the ongoing discussions on the Horizon Europe work programme and call topic(s) with some questions: (1) which type of projects have been approved, is there enough diversity? (2) what are the common results and experiences, are there perhaps similar lessons to be learned from the funding policy viewpoint? (3) it has been identified that technology is critical (more than 50% of recommendations touch this field): How can technology related lessons learned be incorporated into future Topic Calls? Is technology been used in a way prescribed by the call?

As the consortium of the PROCare4Life project, we cannot comment on the previous issues, but only recommend considering them for the preparation of future calls. While the analysis and discussion of these points would be worth a paper of its own, we only would like to state one request here, regarding in particular the third issue, we suggest that any statement on what a technical solution or device is able to provide at the time of entering the project must be very reliable. We can cite in this respect exemplary statements on technology our call "SC1-DTH-11-2019: Large Scale pilots of personalised & outcome based integrated care" contains: Specific Challenge: “Ensure trust of users and policy makers with regard to data access, protection and sharing"; Scope: “to foster the large-scale pilots for deployment of trusted and personalised digital solutions"; Expected outcomes: “Ensuring secure and efficient sharing and processing of all data and information involved in the supply chain”; Expected Impact: “A common vision of technical prerequisites and framework to ensure users trust with regard to health and social data and information in an IT-supported environment

The TRL (technology readiness level) is not sufficient for raising THIS trust. To achieve this reliability, a standardised assessment of Horizon-funded technology development is necessary. The existing evaluation by external monitors and reviewers is fine for commenting the project as such, but reading deliverables and asking questions is not enough for the assessment and feature documentation needed to improve the funding procedures, in order to start a new project based on reliable results of other projects.

## Conclusions

For transferring the lessons-learned into future projects, it is crucial to represent the process from the grant agreement phase (1), which is the foundation for each project, to the realization phase (2), where the plans and objectives need to remain on track.

(1) The multi-disciplinary consortium members need to prepare a concise grant agreement filling the standard sections but on top of it taking care of transparent plans avoiding grey areas, including detailed descriptions of the conditions in each pilot centre, eliminating overlapping of the pilots, listing realistic number of participants in the pilots, and recruitment timelines, adding pre-piloting sessions for technology feasibility tests in lab-environment, but also at a later stage feasibility tests with a handful of participants in the real-environment of each pilot centre, including installation tests of the technologies with non-technical- stuff. For adopting the solution, flexible budgets are essential. Users require technology-training sessions before intervention starts. Dedicated time for dissemination including publications of the researchers need to be incorporated.

(2) During the project a mixture of in-person meetings and weekly regular online meetings with minimum one staff member from each partner should be established. Also, technical guidance with fixed regular meeting slots for questions must be offered. Regarding the technology, a team should be created which monitors and evaluates the market updates and data quality. A common publication procedure is fundamental to inform the project leaders and related team members about the dissemination plans and to promote scientific publications. When closing this article PROCare4Life database analyses was in progress.

(3) The procedures for assessment and documentation of technology development and status at project end should be standardised in more detail, to allow for trustful usage in other projects.

**Disclaimer:** The statements, opinions and data contained in this publication are solely those of the individual author(s) and contributor(s). It reflects only the author's view and that the EU Commission is not responsible for any use that may be made of the information it contains.

## Data Availability

PROCare4Life consortium is under the obligation to protect results because of a legitimate interest of confidentiality under Grant Agreement number 875221.

## Footnotes

^ii^ European Commission. 2021. *The Ageing Report. Economic and Budgetary Projections for the EU Member States (2019-2070).* ISSN 2443-8014 (online), The 2021 Ageing Report. Economic and Budgetary Projections for the EU Member States (2019-2070) (europa.eu)

^iii^ European Commission. 2021. *Green Paper on Ageing. Fostering solidarity and responsibility between generations.* Adopted on 27 January 2021, COM (2021) 50 final. green_paper_ageing_2021_en.pdf (europa.eu)

^iv^ Overview - European Commission (europa.eu)

^v^ Ferreira, F., Almeida, N., Rosa, A. F., Oliveira, A., Casimiro, J., Silva, S., & Teixeira, A. (2014). Elderly Centered Design for Interaction – The Case of the S4S Medication Assistant. Procedia Computer Science, 27, 398–408. https://doi.org/10.1016/j.procs.2014.02.044

^vi^ Duque, E., Fonseca, G., Vieira, H., Gontijo, G., & Ishitani, L. (2019). A systematic literature review on user centered design and participatory design with older people. Proceedings of the 18th Brazilian Symposium on Human Factors in Computing Systems, 1–11. https://doi.org/10.1145/3357155.3358471
Gould, J. D., & Lewis, C. (1985). Designing for usability: key principles and what designers think. Communications of the ACM, 28 (3), 300–311. https://doi.org/10.1145/3166.3170
Kujala, S. (2003). User involvement: A review of the benefits and challenges. Behaviour & Information Technology, 22 (1), 1–16. https://doi.org/10.1080/01449290301782

^vii^ Cozza, M., Tonolli, L., & D’Andrea, V. (2016). Subversive participatory design. Proceedings of the 14 ^th^ Participatory Design Conference: Short Papers, Interactive Exhibitions, Workshops - Volume 2, 53–56. https://doi.org/10.1145/2948076.2948085
Jackson, C. L., & Greenhalgh, T. (2015). Co-creation: a new approach to optimising research impact? Medical Journal of Australia, 203 (7), 283–284. https://doi.org/10.5694/mja15.00219
Schuler, D., & Namioka, A. (1993). Participatory Design (D. Schuler & A. Namioka, Eds.). CRC Press. https://doi.org/10.1201/9780203744338

^viii^ Rowe, S. F. & Sikes, S. (2006). Lessons learned: taking it to the next level. Paper presented at PMI ^®^ Global Congress 2006—North America, Seattle, WA. Newtown Square, PA: Project Management Institute.

^ix^ Kazi, Mashrur & Chowdhury, Nashit & Chowdhury, Mohammad & Turin, Tanvir. (2021). Conducting a rapid review for quick turnaround knowledge synthesis. Health and Primary Care. 5. 2021. 10.15761/HPC.1000213.

^x^ - König A, Hatzakis T, Andrushevich A *et al.* A reflection on participatory research methodologies in the light of the COVID-19 – lessons learnt from the European Research Project TRIPS [version 2; peer review: 2 approved]. Open Res Europe 2022, **1**:153 ( https://doi.org/10.12688/openreseurope.14315.2) - Ospina Y, Aprà FM, Aggeli A *et al.* Lessons learnt during the workshop “Empowerment of citizens: fostering user engagement for innovative demand response for effective flexibility” [version 1; peer review: 3 approved with reservations]. Open Res Europe 2023, 3:1 ( https://doi.org/10.12688/openreseurope.15270.1) - Morgan, H. (2022). Conducting a Qualitative Document Analysis. The Qualitative Report, 27(1), 64–77. https://doi.org/10.46743/2160-3715/2022.5044
- Paleari L, Malini V, Paoli G, Scillieri S, Bighin C, Blobel B and Giacomini M (2022) EU-Funded Telemedicine Projects – Assessment of, and Lessons Learned From, in the Light of the SARS-CoV-2 Pandemic. Front. Med. 9:849998. Doi: 10.3389/fmed.2022.849998
- Fiordelli M, Fadda M, Amati R, Albanese E (2021) Older adults’ motivations to participate or not in epidemiological research. Qualitative inquiry on a study into dementia in Switzerland. PLoS ONE 16(2): e0247141. https://doi.org/10.1371/journal.Pone.0247141
- Sheila Payne, Yakubu Salifu, Dunun Begovic, and Anthony Greenwood. 2022. *The European MyPal Study: Developing digital health for adults and children with cancer.* Lancaster University. Publications | MyPal (mypal-project.eu)
- European Commission success stories website and search engine: All success stories | Research and Innovation (europa.eu); 2017 “Asking Questions About DEAR Projects”: Asking Questions About DEAR Projects | Capacity4dev (europa.eu)
- Raben *et al.* (2021) Lessons learned from the first European project on the integration of infectious diseases in testing services, data collection and country responses, in BMC Infection Diseases, Volume 21 Supplement 2 Results from INTEGRATE - the EU Joint Action on integrating prevention, testing and linkage to care strategies across HIV, viral hepatitis, TB and STIs in Europe
- Liljas AEM, Walters K, Jovicic A, Iliffe S, Manthorpe J, Goodman C, Kharicha K. Strategies to improve engagement of 'hard to reach' older people in research on health promotion: a systematic review. BMC Public Health. 2017 Apr 21;17(1):349. doi: 10.1186/s12889-017-4241-8. PMID: 28431552; PMCID: PMC5399821 - Lettieri E, Fumagalli LP, Radaelli G, Bertele’ P, Vogt J, Hammerschmidt R, Lara JL, Carriazo A, Masella C. Empowering patients through eHealth: a case report of a pan-European project. BMC Health Serv Res. 2015 Aug 5;15:309. Doi: 10.1186/s12913-015-0983-0. PMID: 26242863; PMCID: PMC4526304. - A.R.S. Progetti S.p.a. in consortium with: Lattanzio Advisory S.p.a. and AGRER S.A. N.V. 2017. “Support to enhance livelihoods per people dependent on informal economy and improve social inclusion of marginalised and vulnerable persons” Volume 4.3: Good Practices and Lessons Learned. Case studies of 15 EU funded projects”, bing.com/ck/a?!&&p=f698c3be2e74c549JmltdHM9MTcwNDI0MDAwMCZpZ3VpZD0zMjk4YjY1Yy1mNmI1LTY5ODUtMGExZi1hNWE3Zjc0NjY4YjImaW5zaWQ9NTE3MA&ptn=3&ver=2&hsh=3&fclid=3298b65c-f6b5-6985-0a1f-a5a7f74668b2&psq=“Support+to+enhance+livelihoods+per+people+dependent+on+informal+economy+and+improve+social+Inclusion+of+marginalised+and+vulnerable+persons”+Volume+4.3%3a+Good+Practices+and+Lessons+Learned+Case+studies+of+15+EC-funded+projects&u=a1aHR0cHM6Ly9jYXBhY2l0eTRkZXYuZXVyb3BhLmV1L21lZGlhLzY3Odg5L2Rvd25sb2FkLzE4OGMxOTU4LTk5MGItNGEyYi05MGFmLTdlMTFkMWI2YTM3ZV9lbg&ntb=1
- Iain Corby. 2022. “A summary of the achievements and lessons learned of the euCONSENT project and what comes next”, A summary of the achievements and lessons learned of the euCONSENT project and what comes next - EuConsent
- A multi-stakeholder co-creation platform for better access to Long-Term Care services (SOCATEL) EU funded project. 2021. Deliverable: D.6.4 Learnt lessons and replication guidelines, D6-4.pdf (socatel.eu); DRIVER+ EU funded project. 2020. D911.91-Lessons Learned on Project Level. SP91. Project Management, DRIVER_D911.91_Lessons-learned-on-project-level.pdf (driver-project.eu)
- eConfidence EU funded project. 2017. “DEL. 7.5 Report with lessons learnt from other projects and initiatives. Practical implementation plan for undertaking the data collection and analysis activities”, https://ec.europa.eu/research/participants/documents/downloadPublic?documentIds=080166e5b200a26b&appId=PPGMS;
