# Supplementary material for: PROCare4Life lessons learned
Source: Open Res Eur. 2026 May 13;3:147. Originally published 2023 Sep 11. [Version 3] doi: 10.12688/openreseurope.16304.3 (PMC11019287; doi:10.12688/openreseurope.16304.3)
Supplement: Peer review history

# Reviewer report: Reviewer response for version 2

**DOI:** 10.21956/openreseurope.18581.r39529

**Published:** 2024-06-12

**Authors:** Raphael Wittenberg, Jacqueline Damant

**Affiliations:** 1 London School of Economics and Political Science, London, UK

This paper presents lessons learned from the PROCare4Life project and the methodology used to identify them. The challenges identified and recommendations set out in the paper will be valuable for future projects.

 While the aim of the paper is stated clearly in the abstract, it is less clear in the plain English summary and the introduction section. Much of the plain English summary focuses on the term implementation and no mention is made of methods used to identify the challenges and recommendations. The introduction does not mention the aim of the paper until the final three sentences, and those sentences are not entirely clear.

 The methods section is not easy to follow and raises some questions. It is unclear how the different methods outlined in Figure 1 and described in the associated text relate to each other or to the pilots discussed later in the paper. More specifically:

 Method 2: Did this involve documentation of material that had not previously been documented or did it involve search for and analysis of material in existing documents?

 Method 4: Are the meetings mentioned under 4 the same as those mentioned under 3? Was the method 4 activity wholly separate from and prior to activities under 1 to 3?

 Method 5: Were consortium members participating in the questionnaire sent a draft list of challenges and/or recommendations or were they asked the questions without being presented with a draft list? Would they have drawn on the findings from activities under 1 to 4? What was the response rate to the questionnaire?

 Method 6: Did this inform categorising challenges and recommendations from lists that had been finalised after activity 5?

 Method 7: Does this mean that the challenges and recommendations presented in Figures 3 and 4 and/or those in tables 5 to 7 are in priority order?

 Pilots: Did their findings contribute to any of the processes 1 to 7?

  I cannot state that I have expertise in all the methods listed in Figure 1 and outlined in the accompanying text. I cannot be certain about the level of scientific standard of the work described in the paper. I think it would be acceptable subject to clarifications as per our comments above.

Is the case presented with sufficient detail to be useful for teaching or other practitioners?

Partly

Is the work clearly and accurately presented and does it cite the current literature?

Partly

If applicable, is the statistical analysis and its interpretation appropriate?

Not applicable

Are all the source data underlying the results available to ensure full reproducibility?

No

Are the conclusions drawn adequately supported by the results?

Yes

Is the background of the case’s history and progression described in sufficient detail?

Yes

Reviewer Expertise:

Economics of long-term care, including care for people with dementia

We confirm that we have read this submission and believe that we have an appropriate level of expertise to confirm that it is of an acceptable scientific standard, however we have significant reservations, as outlined above.

# Reviewer report: Reviewer response for version 2

**DOI:** 10.21956/openreseurope.18581.r39106

**Published:** 2024-04-15

**Authors:** Alvaro Sanchez Ferro

**Affiliations:** 1 Servicio de Neurología, Hospital Universitario 12 de Octubre, Madrid, Spain

I am fine with the revisions!

Is the case presented with sufficient detail to be useful for teaching or other practitioners?

Yes

Is the work clearly and accurately presented and does it cite the current literature?

Yes

If applicable, is the statistical analysis and its interpretation appropriate?

Yes

Are all the source data underlying the results available to ensure full reproducibility?

Yes

Are the conclusions drawn adequately supported by the results?

Yes

Is the background of the case’s history and progression described in sufficient detail?

Yes

Reviewer Expertise:

NA

I confirm that I have read this submission and believe that I have an appropriate level of expertise to confirm that it is of an acceptable scientific standard.

# Reviewer report: Reviewer response for version 2

**DOI:** 10.21956/openreseurope.18581.r39105

**Published:** 2024-04-15

**Authors:** Cristina Nombela Otero

**Affiliations:** 1 Departamento de Psicología Biológica y de la Salud, Autonomous University of Madrid, Madrid, Community of Madrid, Spain

The document has undergone substantial improvement. The suggestions I proposed weeks ago have been incorporated, along with additional revisions that offer a significant and valuable contribution to the field.

Is the case presented with sufficient detail to be useful for teaching or other practitioners?

Partly

Is the work clearly and accurately presented and does it cite the current literature?

Partly

If applicable, is the statistical analysis and its interpretation appropriate?

Not applicable

Are all the source data underlying the results available to ensure full reproducibility?

Partly

Are the conclusions drawn adequately supported by the results?

Yes

Is the background of the case’s history and progression described in sufficient detail?

Yes

Reviewer Expertise:

Cognitive scientists

I confirm that I have read this submission and believe that I have an appropriate level of expertise to confirm that it is of an acceptable scientific standard.

# Reviewer report: Reviewer response for version 1

**DOI:** 10.21956/openreseurope.17601.r35715

**Published:** 2023-11-22

**Authors:** Alvaro Sanchez Ferro

**Affiliations:** 1 Servicio de Neurología, Hospital Universitario 12 de Octubre, Madrid, Spain

Gangas et al. present a summary of the lessons learned during an EU-funded project, PROCare4Life. In this project, different digital technologies are integrated and evaluated using codesign methodologies. They included a significant number of users including various neurodegenerative conditions (dementia, Parkinson's disease) as well as healthy elders. The authors used what is called mixed-methods research to evaluate their learnings and present these results that can be very interesting for future projects developed in this space.

 With respect to possible improvements, the authors could consider a text revision might be beneficial to avoid lengthy sentences, spelling out acronyms as well, and having a native speaker review that the content is correct. 

 The other potential improvement will be to clarify how the previous body of evidence was compared to the project learnings, it will be helpful to include more details about the internet search (flowchart of the results, keywords, etc) and also how this helped craft or shape the lessons learned described in the work.

Is the case presented with sufficient detail to be useful for teaching or other practitioners?

Yes

Is the work clearly and accurately presented and does it cite the current literature?

Yes

If applicable, is the statistical analysis and its interpretation appropriate?

Yes

Are all the source data underlying the results available to ensure full reproducibility?

Yes

Are the conclusions drawn adequately supported by the results?

Yes

Is the background of the case’s history and progression described in sufficient detail?

Yes

Reviewer Expertise:

Neurology, Technology, Diagnostics, Biomedical Research

I confirm that I have read this submission and believe that I have an appropriate level of expertise to confirm that it is of an acceptable scientific standard, however I have significant reservations, as outlined above.

# Response

**Published:** 2024-01-23

**Authors:** PILAR GANGAS

**Affiliations:** Scientific Advise Unit, Consejo Superior de Investigaciones Cientificas, Madrid, Spain, Spain

Thank you so much for your comments and suggestions, that have been very valuable for improving the previous version of the article.  As suggested, a native speaker, our colleague David Linnane, political scientist and journalist of IFIC, has revised the article to address this suggestion.  As requested, additional information has been provided on the review process and its results. A methods processual approach flowchart has been included, including keywords and describing more in detail how this helped shape the lessons learned described in the work.

# Reviewer report: Reviewer response for version 1

**DOI:** 10.21956/openreseurope.17601.r35173

**Published:** 2023-10-19

**Authors:** Cristina Nombela Otero

**Affiliations:** 1 Departamento de Psicología Biológica y de la Salud, Autonomous University of Madrid, Madrid, Community of Madrid, Spain

The manuscript by Gangas et al describes the lessons learnt from the EU funded project PROCare4life. The content is highly valuable, and the tables provided a well organized way to perceive the key elements for patients/relatives/and other users involved in chronic disorders environment.

 I just provide some suggestions for making the reading more enriching for the readers.

**Abstract**

 The term “implementation” is used in this section and at the beginning of the Introduction section in a not completely clear way. The beginning of the description of the project starts with the term “implementation”, without providing a description of the design phase. Actually, implementation regards the “execution or practice of a plan, a method or any design, idea, model, specification, standard or policy for doing something. As such, implementation is the action that must follow any preliminary thinking for something to actually happen“ (Ben Lutkevich, 2022). As such, the use provided for “implementation” leads to thinking that there is no design phase, what doesn´t seem to be the case. I suggest reviewing the use of the term “implementation” along the document.

**Introduction**

 The Introduction doesn´t describe the state of the art that sustained the idea of this project. Instead of that, the intro includes a general description of the steps taken, together with some aspects of the methods (what should go into the “method section”). E.g. in page 4 appears, “Over PROCare4Life implementation, three waves of pilots in six pilots’ sites helped to codesign, fine-tune and improve the PROCare4Life system. The pilot sites have been the following: Spitalul Universitar de Urgenta Bucuresti (Bucharest); Association Parkinson Madrid (Madrid); Casa di Cura Igea (Milan); Campus Neurológico Senior (Lisbon); University of Medicine and Pharmacy (Bucharest); Wohlfahrtswerk für Baden-Württemberg (Stuttgart)”. In my opinion, the list of pilot sites goes into the Methods section instead of appearing into the Introduction section. A better organization of the content would help readers to localized the relevant info for each of them, getting the most of the knowledge generated at the PROCare4Life project.

**Methods**

 The first paragraph is devoted to the lessons learnt from the project, instead of describing the methods followed to reach the results. Later, once the results are provided, it would be the time to describe the lessons provided. Actually, the “lessons learnt” would meet the content of the “Conclusions section” (in my view). In the subsequent paragraphs of “Methods” section there is a brief description of the methods used (biweekly meetings, Iterative qualitative method,…). However, the methods followed should be described according to any organizational criteria (either temporal -explaining the order followed in the methods used- or spatial criteria – explaining the processes followed at each pilot site, for example). A good idea might be to provide details on the application of such methods and the content managed at each phase of the project. That would help the readers to understand and adapt the methods and techniques used. Also, the number of participants at each site/phase of the study could be provided.

**Results**

 The objectives are named at the beginning of the results section, instead of doing it at the end of the introduction or at the beginning of the Methods section.

 In general terms, I suggest organizing each section making a difference between: 1) the organization of the project from the perspective of the entities involved, and b) the perspective of the end users who participated.

**Tables**

 That´s probably one of the biggest points of the manuscript since the information is really well organized and nicely explained on the tables.

Is the case presented with sufficient detail to be useful for teaching or other practitioners?

Partly

Is the work clearly and accurately presented and does it cite the current literature?

Partly

If applicable, is the statistical analysis and its interpretation appropriate?

Not applicable

Are all the source data underlying the results available to ensure full reproducibility?

Partly

Are the conclusions drawn adequately supported by the results?

Yes

Is the background of the case’s history and progression described in sufficient detail?

Yes

Reviewer Expertise:

Cognitive scientists

I confirm that I have read this submission and believe that I have an appropriate level of expertise to confirm that it is of an acceptable scientific standard, however I have significant reservations, as outlined above.

# Response

**Published:** 2024-01-23

**Authors:** PILAR GANGAS

**Affiliations:** Scientific Advise Unit, Consejo Superior de Investigaciones Cientificas, Madrid, Spain, Spain

Thank you so much for the detailed reading of the article and the valuable suggestions, that we have carefully considered and revised the first version of th article accordingly.  To respond to the first comments on the abstract section, a definition of implementation has been included in that section to clarify this point and what is referred to when using the term "implementation" by PROCare4Life team.  In the Introduction section, a State of the Art, connecting the project goals with the EU Digital Strategy has been included. The referred sentences have been deleted from the introduction and included in the methods section, as suggested. In the methods section, lessons learned methodology has been introduced in the first sentence of the paragraph. Processual approach has been used for the methodology, which has been specified in the article. Although lessons learned research has been implemented by the consortium all over the project phases, only the lessons learned researched under pilot 3 are described in this article.  In the results, the objectives have been relocated to the introduction section. The results presented in this article are based solely on the contribution of the PROCare4Life consortium members. Final end users have not participated directly in this process, although their feedback has been considered by the research team. This clarification has been included in the article.
