# Supplementary material for: PROCare4Life lessons learned
Source: Open Res Eur. 2024 Mar 22;3:147. Originally published 2023 Sep 11. [Version 2] doi: 10.12688/openreseurope.16304.2 (PMC11019287; doi:10.12688/openreseurope.16304.2)
Supplement: Peer review history

# Response

**Published:** 2024-01-23

**Authors:** PILAR GANGAS

**Affiliations:** INTERNATIONAL FOUNDATION FOR INTEGRATED CARE, Spain

Thank you so much for your comments and suggestions, that have been very valuable for improving the previous version of the article.  As suggested, a native speaker, our colleague David Linnane, political scientist and journalist of IFIC, has revised the article to address this suggestion.  As requested, additional information has been provided on the review process and its results. A methods processual approach flowchart has been included, including keywords and describing more in detail how this helped shape the lessons learned described in the work.

# Response

**Published:** 2024-01-23

**Authors:** PILAR GANGAS

**Affiliations:** INTERNATIONAL FOUNDATION FOR INTEGRATED CARE, Spain

Thank you so much for the detailed reading of the article and the valuable suggestions, that we have carefully considered and revised the first version of th article accordingly.  To respond to the first comments on the abstract section, a definition of implementation has been included in that section to clarify this point and what is referred to when using the term "implementation" by PROCare4Life team.  In the Introduction section, a State of the Art, connecting the project goals with the EU Digital Strategy has been included. The referred sentences have been deleted from the introduction and included in the methods section, as suggested. In the methods section, lessons learned methodology has been introduced in the first sentence of the paragraph. Processual approach has been used for the methodology, which has been specified in the article. Although lessons learned research has been implemented by the consortium all over the project phases, only the lessons learned researched under pilot 3 are described in this article.  In the results, the objectives have been relocated to the introduction section. The results presented in this article are based solely on the contribution of the PROCare4Life consortium members. Final end users have not participated directly in this process, although their feedback has been considered by the research team. This clarification has been included in the article.
